# Evolution of Apathy in Early Parkinson's Disease: A 4-Years Prospective Cohort Study

**DOI:** 10.3389/fnagi.2020.620762

**Published:** 2021-01-28

**Authors:** Ruwei Ou, Junyu Lin, Kuncheng Liu, Zheng Jiang, Qianqian Wei, Yanbing Hou, Lingyu Zhang, Bei Cao, Bi Zhao, Wei Song, Huifang Shang

**Affiliations:** Department of Neurology, Laboratory of Neurodegenerative Disorders, National Clinical Research Center for Geriatrics, West China Hospital, Sichuan University, Chengdu, China

**Keywords:** Parkinson's disease, apathy, depression, executive function, cohort study

## Abstract

**Objective:** We investigated the prevalence, evolution, associated factors, and risk factors of apathy in a cohort of patients with early-stage Parkinson's disease (PD), who underwent a 4-years prospective follow-up.

**Methods:** This study included 188 patients with PD (baseline disease duration <3 years) who underwent an annual evaluation using the Lille Apathy Rating Scale (LARS). Based on the cut-off value of −21 observed on the LARS, patients were categorized as PD with and without apathy. The generalized estimating equations (GEE) model was utilized to determine the factors associated with apathy, and the Cox proportional-hazards regression model was used to determine the predictors of apathy.

**Results:** Apathy increased from a baseline rate of 18.6–28.8% after 4 years; notably, this rate was not persistent across patients' visits. The LARS score was independently associated with the male sex (B 8.131, *p* = 0.009), low Frontal Assessment Battery (FAB) scores (B 0.567, *p* = 0.011), low attention scores on the Montreal Cognitive Assessment (MOCA) test (B 0.217, *p* = 0.026), high Hamilton Depression Rating Scale (HDRS) scores (B 1.362, *p* < 0.001), high Unified Parkinson's Disease Rating Scale (UPDRS) part III scores (B 1.147, *p* < 0.001), and prolonged follow-up time (B 1.785, *p* = 0.048). A high HDRS score was the only predictor of apathy in PD [hazard ratio (HR) 1.043, *p* = 0.026].

**Conclusions:** The risk of apathy is higher in men with progressive PD accompanied by disease-specific motor and non-motor symptoms. Depression during early-stage PD is a primary risk factor for apathy in PD.

## Introduction

Apathy, a common neuropsychiatric symptom in patients with Parkinson's disease (PD), can occur both in the early and advanced stages of PD and may even precede the motor symptoms of the disease (Pagonabarraga et al., 2015). Apathy is a non-motor symptom that refers to a state of reduced motivation that manifests as diminished goal-directed behaviors, reduced interests, or emotional features that cannot be attributed to altered levels of consciousness, cognitive impairment, or emotional distress (Marin, 1991). Reportedly, apathy is associated with older age, cognitive impairment, depression, and more severely disabling disease in patients with PD (Pagonabarraga et al., 2015), which can negatively affect the quality of life of patients (Gerritsen et al., 2005) and increase the burden of the caregiver (Martinez-Martin et al., 2015).

Most previous studies on apathy in PD have used cross-sectional designs. Although apathy is commonly observed in patients with PD, its prevalence varies significantly (ranging from 16.5 to 60%; Den Brok et al., 2015). To date, few prospective studies with a repeated measures design have focused on apathy in PD. Most studies have included a short follow-up period or a small sample size (Pedersen et al., 2009; Santangelo et al., 2015b; Wee et al., 2016). Therefore, limited data are available regarding the long-term evolution and trajectory of apathy in patients with early-stage PD; further research is warranted to gain a deeper understanding of the associated and predicted factors of apathy in patients with PD. This information would benefit clinicians in real-world practice because apathy is often associated with poor prognosis (Starkstein et al., 2006) and poor response to treatment (Mega et al., 1999).

We prospectively investigated patients with early-stage PD, who underwent a 4-years follow-up to determine the prevalence, evolution, associated factors, and risk factors of apathy in patients with PD.

## Methods

### Study Design and Population

This study is a part of an ongoing long-term prospective longitudinal cohort study performed at the Department of Neurology, West China Hospital, Sichuan University, to investigate the progression and prognosis of PD in Chinese patients (*n* = 302). This project initiated in February 2014 and aimed to recruit patients with early PD (disease duration <3 years) to a follow-up of at least 10 years. PD was diagnosed based on the United Kingdom Parkinson's Disease Society Brain Bank's clinical diagnostic criteria for PD (Hughes et al., 1992). We excluded patients with dementia, patients with motor complications (including motor fluctuation and dyskinesia), and patients with Hoehn and Yahr (H&Y) stage ≥3 at baseline.

All patients underwent standardized examinations and regular assessments by trained neurologists in our movement disorder center annually. In the present study, the data analysis was performed based on the assessments at baseline, as well as a 1-, 2-, 3-, and 4-years follow-up. Of the 302 patients recruited, 114 were excluded owing to lack of data regarding the assessment of baseline apathy (*n* = 109) or missing data from other assessments at follow-up visits (*n* = 5); therefore, only 188 patients were eligible for inclusion in the study. All included participants completed the baseline and 1-year follow-up visits. The number of patients who lost contact or withdrew informed consent during 2-, 3-, and 4-years follow-up were 5, 63, and 81, respectively. One patient died between 2- and 3-years follow-up and 5 patients died between 3- and 4-years follow-up. In addition, 49 patients had not yet reached the time to finish the 4-years follow-up visit before we carried out the data analysis. Therefore, the number of patients we included at baseline, 1-, 2-, 3-, and 4-years were 188, 188, 183, 124, and 52, respectively.

The study was approved by the Ethics Committee of West China Hospital, Sichuan University, and written informed consent was obtained from all patients.

### Evaluation Protocol

Baseline demographic data and clinical characteristics, including sex, age, age of disease onset, disease duration, and years of schooling, were collected, and the therapeutic regimen was recorded at each visit. Antiparkinsonian medications of patients were converted into the total levodopa equivalent daily doses (LEDD) based on a previous report (Tomlinson et al., 2010). All patients with PD underwent baseline and periodic neurological evaluation during the follow-up. The severity of motor symptoms associated with PD was evaluated using the Unified Parkinson's Disease Rating Scale (UPDRS) part III (Movement Disorder Society Task Force on Rating Scales for Parkinson's Disease, 2003) and the H&Y stage (Hoehn and Yahr, 1967). The Hamilton Depression Rating Scale (HDRS) containing 24 items (Moberg et al., 2001) was used to evaluate depression, and the Hamilton Anxiety Rating Scale (HADS; Hamilton, 1959) was used to evaluate anxiety. The executive function was evaluated using the Frontal Assessment Battery (FAB; Dubois et al., 2000). The Montreal Cognitive Assessment (MOCA) screening tool was used for global cognitive function evaluation (Nasreddine et al., 2005). The MOCA contains the following seven subdomains: visuospatial/executive ability, naming, attention, language, abstraction, memory, and orientation.

### Apathy Evaluation

Apathy was evaluated annually using the Lille Apathy Rating Scale (LARS) (Leentjens et al., 2008), a validated scale to assess apathy in PD. The total LARS score ranges between −36 and +36, with cut-off values for non-apathy, slight apathy, moderate apathy, and severe apathy being (−36 to −22), (−21 to −17), (−16 to −10), and (−9 to +36), respectively. The prevalence of apathy observed at each visit was calculated based on the percentage of patients with a LARS score of ≥-21 (Leentjens et al., 2008).

### Statistical Analyses

All statistical analyses were performed using SPSS software, version 22.0, or R software, version 4.0.2. All statistical tests were two-tailed, and the values of *p* < 0.05 were considered statistically significant. Continuous variables are represented as means and SD, and categorical variables as counts and percentages. The chi-square test, the Fisher's exact test, and the Student's *t-*test were used for an intergroup comparison of the clinical variables.

Population-averaged regression models using generalized estimating equations (GEE) with multiple linear regression analysis were used to determine the factors associated with the severity of apathy. The models were based on all patients in the cohort, included all consecutive examinations over the follow-up period, and allowed for a correlation between repeated measurements obtained in the same patients. An exchangeable working correlation structure was selected. The dependent variable, the LARS score, was used as a continuous variable in the model. The independent variables included the following repeated measures: age, sex, disease duration, education level, the LEDD, the administration of levodopa, the administration of dopamine agonists, the administration of antidepressants, the UPDRS part III score, the H&Y stage, the FAB score, the total MOCA score along with its subdomain scores, the HDRS score, the HARS score, and the follow-up time in years. The GEE analysis was first performed using only a single covariate at a time (unadjusted model) and was subsequently performed using all covariates that showed the values of *p* < 0.1 or were possibly associated with apathy (adjusted model).

The univariate and multivariate Cox proportional-hazards regression models were used to determine the predictors of apathy in PD. These models were used for the evaluation of patients who reported the absence of baseline apathy. The clinical outcome was defined as an onset of apathy (both persistent and non-persistent) observed during the follow-up. The univariate analysis (unadjusted model) included only a single covariate at a time. The multivariate analysis (adjusted model) included the following variables: sex, the FAB score, the HDRS score, and the UPDRS III score based on the values of *p* < 0.1 or a probable association with apathy based on clinical judgment. The Schoenfeld individual test was used to determine the proportional hazard assumption, where a value of *p* > 0.05 indicated that the data met the criteria.

### Data Availability

Anonymized data can be obtained upon request from qualified investigators for the purposes of replicating procedures and results.

## Results

### Baseline and Follow-Up Data

No statistically significant differences were observed in the baseline characteristics between patients who were included in the current study (*n* = 188) and those who did not (*n* = 114; Supplementary Table 1). The demographic and clinical features of patients with PD included in this study are listed in Table 1. We included 188 patients with PD at baseline (50.5% men). The mean patient age at baseline was 58.1 (SD 10.7) years, with mean disease duration of 1.5 (SD 0.8) years. The LARS score increased from −27 (SD 10.4) at baseline to −25 (SD 9.4) after 4 years. Baseline antiparkinsonian therapy was administered to 90 patients (47.9%), and this figure increased to 100% after 4 years, with a mean increase in the LEDD from 152.5 mg/day (SD 188.1) to 529.8 mg/day (SD 210.7).

**Table 1:** Demographic and clinical features of PD patients.

	**Baseline**	**1-year**	**2-years**	**3-years**	**4-years**
Number of samples	188	188	183	124	52
Age, years, mean (SD)	58.1 (10.7)	59.3 (10.7)	60.7 (10.7)	61.2 (11.1)	63.6 (10.4)
Disease duration, mean (SD)	1.5 (0.8)	2.7 (1.0)	4.1 (1.1)	5.2 (1.2)	6.0 (1.1)
Male sex, *n* (%)	95 (50.5)	95 (50.5)	93 (50.8)	62 (50.0)	31 (59.6)
Education, mean (SD)	10.7 (4.1)	10.7 (4.1)	10.7 (4.1)	10.7 (4.2)	10.6 (4.0)
LEDD, mg/day, mean (SD)	152.5 (188.1)	329.9 (174.9)	430.8 (195.7)	537.5 (211.6)	529.8 (210.7)
Use of levodopa, *n* (%)	74 (39.4)	128 (68.1)	148 (80.9)	113 (91.1)	50 (96.2)
Use of dopamine agonist, *n* (%)	46 (24.5)	124 (66.0)	152 (83.1)	110 (88.7)	47 (90.4)
Use of anti-depressant, *n* (%)	8 (4.3)	6 (3.2)	17 (9.3)	12 (9.7)	4 (7.7)
Use of AChE-I, *n* (%)	0	0	0	1 (0.8)	1 (1.9)
FAB score, mean (SD)	16.4 (1.9)	16.3 (2.2)	16.3 (2.0)	16.4 (1.8)	16.3 (2.1)
MOCA score, mean (SD)	25.5 (3.5)	26.0 (3.5)	25.8 (3.5)	25.8 (3.6)	25.8 (3.7)
HDRS score, mean (SD)	7.8 (7.2)	7.2 (6.4)	7.3 (6.4)	7.6 (5.9)	8.0 (6.1)
HARS score, mean (SD)	5.6 (5.5)	6.2 (6.0)	6.0 (5.3)	6.2 (5.3)	7.0 (5.7)
UPDRS III score, mean (SD)	23.3 (10.6)	25.4(10.6)	27.5 (10.7)	29.1 (11.9)	31.2 (10.1)
H&Y, mean (SD)	1.9 (0.4)	2.0 (1.8)	2.1 (0.5)	2.2 (0.5)	2.2 (0.4)
LARS, mean (SD)	−27.0 (10.4)	−27.3 (7.6)	−26.1 (9.2)	−25.5 (8.8)	−25.0 (9.4)

*PD, Parkinson's disease; LEDD, levodopa equivalent daily dose; AChE-I, acetylcholinesterase inhibitor; FAB, frontal assessment battery; MOCA, Montreal Cognitive Assessment; HDRS, Hamilton Depression Rating Scale; HARS, Hamilton Anxiety Rating Scale; UPDRS, Unified Parkinson's Disease Rating Scale; LARS, Lille Apathy Rating Scale*.

### Prevalence and Evolution of Apathy

**Figure 1:**
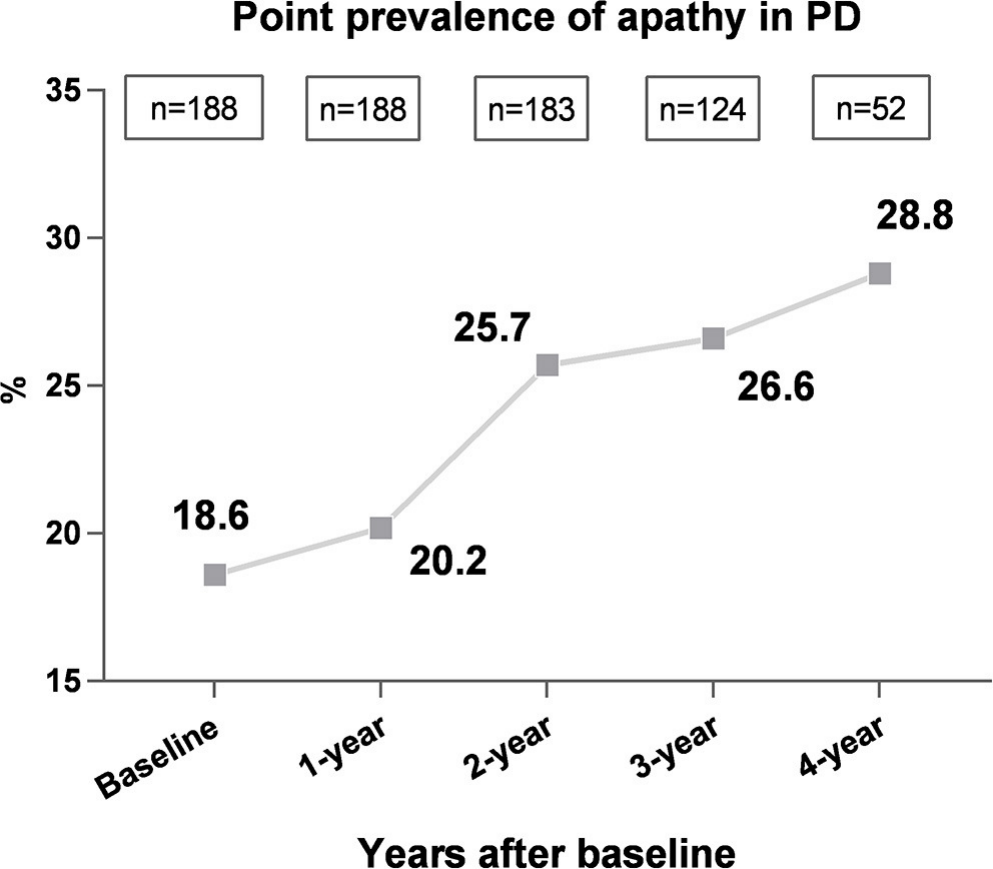
Frequency of apathy over time in patients with PD. The prevalence of apathy in patients with PD increased with disease duration over time, which increased from 18.6% at baseline to 28.8% after 4 years.

Figure 1 shows the observed point prevalence of apathy in patients with PD. Of the 188 patients, 35 (18.6%) had apathy at baseline. During the follow-up, we observed that the 1-, 2-, 3-, and 4-years prevalence rates increased to 20.2% (38/188), 25.7% (47/183), 26.6% (33/124), and 28.8% (15/52), respectively.

**Figure 2:**
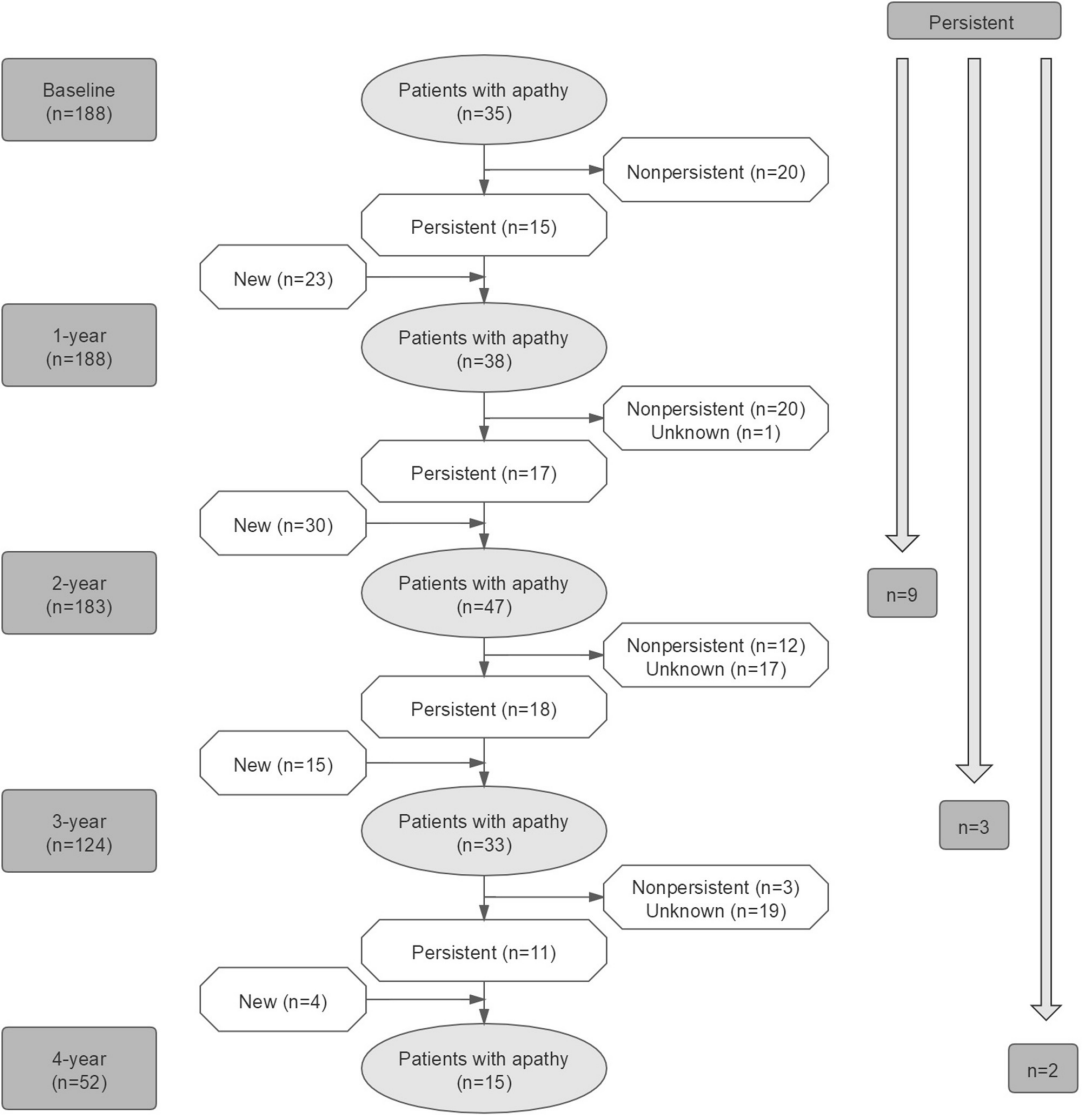
Evolution of apathy in patients with PD over time.

We observed that in most patients with PD, apathy was not persistent across visits during the 4-years study period (Figure 2). Nine patients with PD showed persistent apathy after 2 years, whereas only three patients and two patients showed persistent apathy after 3 and 4 years, respectively.

A total of 52 patients completed the 4-years follow-up. No significant difference was observed in the baseline characteristics between patients with and without complete 4-years follow-up (Supplementary Table 2).

### Factors Associated With Apathy in Patients With PD Over Time

At baseline, patients with apathy had a significantly lower score in the FAB (*p* = 0.005) and higher scores in the HDRS (*p* < 0.001), HARS (*p* = 0.007), and UPDRS III (*p* = 0.004; Supplementary Table 3).

Table 2 shows the factors associated with the LARS score in PD over time. The GEE analyses indicated that the LARS score was independently associated with the male sex [B 8.131, 95% confidence interval (CI) 1.673–39.521, *p* = 0.009], low FAB scores (B 0.567, 95% CI 0.366–0.879, *p* = 0.011), low attention subscores on the MOCA screening test (B 0.217, 95% CI 0.056–0.833, *p* = 0.026), high HDRS scores (B 1.362, 95% CI 1.176–1.577, *p* < 0.001), high UPDRS part III scores (B 1.147, 95% CI 1.064–1.236, *p* < 0.001), and prolonged follow-up time (B 1.785, 95% CI 1.005–3.168, *p* = 0.048; adjusted model).

**Table 2:** Factors associated with higher LARS scores in patients with PD.

	**Unadjusted model**			**Adjusted model**		
	**B**	**95%CI**	***P*-value**	**B**	**95%CI**	***P*-value**
Age	1.022	0.927–1.126	0.662	0.946	0.875–1.022	0.160
Disease duration	1.404	0.961–2.053	0.080			
Male sex	7.104	1.172–43.051	0.033*	8.131	1.673–39.521	0.009*
Education	0.767	0.606–0.970	0.027*	0.944	0.784–1.137	0.542
LEDD	1.003	1.000–1.006	0.049*	0.997	0.994–1.000	0.055
Use of levodopa	2.923	0.730–11.711	0.130			
Use of dopamine agonist	1.936	0.436–8.584	0.385			
Use of antidepressant	29.859	0.670–1329.944	0.080	6.360	0.196–206.753	0.298
FAB	0.462	0.286–0.746	0.002*	0.567	0.366–0.879	0.011*
MOCA	0.626	0.482–0.812	<0.001*			
Visuospatial/executive ability	0.365	0.199–0.671	0.001*			
Naming	0.428	0.118–1.558	0.198			
Attention	0.100	0.024–0.420	0.002*	0.217	0.056–0.833	0.026*
Language	0.348	0.125–0.969	0.043*	0.726	0.305–1.730	0.470
Abstraction	0.916	0.310–2.701	0.873			
Memory	0.785	0.468–1.316	0.359			
Orientation	0.114	0.022–0.575	0.009*	0.582	0.148–2.287	0.483
HDRS	1.409	1.248–1.591	<0.001*	1.362	1.176–1.577	<0.001*
HARS	1.329	1.153–1.532	<0.001*	0.959	0.784–1.174	0.688
UPDRS III	1.232	1.138–1.335	<0.001*	1.147	1.064–1.236	<0.001*
H&Y	57.108	8.785–371.264	<0.001*			
Follow-up time in years	1.734	1.040–2.892	0.035*	1.785	1.005–3.168	0.048*

*PD, Parkinson's disease; LARS, Lille Apathy Rating Scale; LEDD, levodopa equivalent daily dose; FAB, frontal assessment battery; MOCA, Montreal Cognitive Assessment; HDRS, Hamilton Depression Rating Scale; HARS, Hamilton Anxiety Rating Scale; UPDRS, Unified Parkinson's Disease Rating Scale*.

* *Significant difference*.

To explore the effect of drugs on the conversion of apathy, we further compared the change in LEDD between patients with and without persistent apathy in PD. At four stages (baseline to 1-year, 1- to 2-years, 2- to 3-years, and 3- to 4-years), the change in LEDD was not significantly different (Supplementary Table 4).

### Predictors of Apathy in Patients With PD

Table 3 shows the risk factors for apathy in PD. The Cox proportional-hazards regression model indicated that a higher HDRS score was the only predictor of apathy in PD [hazard ratio (HR) 1.043, 95% CI 1.005–1.081, *p* = 0.026; adjusted model]. Male sex, baseline FAB score, and baseline UPDRS part III scores were not associated with the development of apathy in PD. The Schoenfeld individual test indicated that the Schoenfeld residuals were not significantly associated with time (*p* = 0.094), suggesting that the Cox model met the proportional hazard assumption (Supplementary Figure 1).

**Table 3:** Predicted factors for the development of apathy in patients with PD (*n* = 153).

	**Unadjusted model**			**Adjusted model**		
	**HR**	**95%CI**	***P*-value**	**HR**	**95%CI**	***P*-value**
Age	0.987	0.960–1.014	0.345			
Female sex	0.718	0.433–1.190	0.198	0.729	0.438–1.213	0.224
Education	0.985	0.928–1.045	0.609			
FAB	0.908	0.820–1.007	0.066	1.004	0.904–1.116	0.937
MOCA	0.928	0.879–0.979	0.006*			
Visuospatial/executive ability	0.855	0.717–1.020	0.082			
Naming	0.746	0.506–1.099	0.139			
Attention	0731	0.546–0.980	0.036*	0.795	0.563–1.124	0.194
Language	0.718	0.539–0.956	0.023*	0.794	0.587–1.076	0.137
Abstraction	0.942	0.687–1.290	0.708			
Memory	0.935	0.789–1.108	0.440			
Orientation	0.879	0.644–1.201	0.418			
HDRS	1.046	1.006–1.087	0.024*	1.043	1.005–1.081	0.026*
HARS	1.030	0.978–1.085	0.264			
UPDRS III	1.026	1.003–1.049	0.026*	1.016	0.994–1.038	0.157
H&Y	1.660	1.173–2.350	0.004*			

*PD, Parkinson's disease; FAB, frontal assessment battery; MOCA, Montreal Cognitive Assessment; HDRS, Hamilton Depression Rating Scale; HARS, Hamilton Anxiety Rating Scale; UPDRS, Unified Parkinson's Disease Rating Scale*.

* *Significant difference*.

## Discussion

In this prospective longitudinal study, we observed an increased prevalence of apathy over time (from 18.6 to 28.8%) in patients with PD; however, apathy was not persistent. We also observed that the severity of apathy was associated with the male sex, the severity of motor symptoms, attention deficits, executive dysfunction, and depression in patients with early PD. Interestingly, the severity of depression was the only predictor for the onset of apathy in patients with PD. Our results highlight that apathy is an early, common, and non-persistent non-motor symptom in patients with early PD and may, therefore, have implications for clinical management.

Although numerous studies have investigated the prevalence of and factors associated with apathy in patients with PD, most of these were cross-sectional studies. Reportedly, the prevalence of apathy ranges between 14 and 40% in patients with early drug-naïve PD (Aarsland et al., 2009; Pedersen et al., 2010; Dujardin et al., 2014; Santangelo et al., 2015b; Liu et al., 2017), which could perhaps be attributed to the differences in study designs, particularly the differences in the definition of apathy, which was assessed by either the LARS, the neuropsychiatric inventory, or a self-reported version of the Apathy Evaluation Scale.

In the current study, the prevalence of apathy increased with disease progression; however, the symptom was not persistent. At the 4-years follow-up, we observed a 1.5-fold increase in the prevalence of apathy among patients with PD, although the overall prevalence was relatively low during the early stage (<30%). Our findings indicate that apathy is one of several major neuropsychiatric symptoms experienced by patients with early PD. The non-persistent property of apathy increases the difficulty to predict its development. The limited sample of patients with persistent apathy in our cohort also contributes to the impossibility to analyze the predictors of persistent apathy in the current study. Further, larger sample studies with the stratified method are needed to clarify the determinants of apathy. Moreover, the association observed between a high LARS score and a prolonged follow-up time in years indicated that the severity of apathy was likely to increase with disease duration, suggesting that the severity of apathy increases with disease progression in patients with PD.

In our study, apathy was more severe among patients with more severe motor disability; this finding is consistent with the results reported by two previous cross-sectional studies (Pedersen et al., 2010; Dujardin et al., 2014) and also supports our prior findings (Liu et al., 2017). These results imply that the dysfunction of the dopaminergic system is a likely contributor to the onset of apathy in PD, which was verified by a previous study using a single-photon emission CT (Santangelo et al., 2015a). These authors reported that after adjusting for age, disease duration, the site of onset of motor symptoms, and the severity of motor symptom, the striatal levels of dopamine transporter were lower in untreated patients with PD with apathy than in those without apathy. Pharmacological studies (Czernecki et al., 2002; Thobois et al., 2013) that reported an improvement in apathy following dopaminergic treatment also partly support the role of dopaminergic deficit in the development of apathy in PD. However, we found that the LEDD changes were not different between patients with and without persistent apathy, and no association was observed between the LEDD and the LARS score, suggesting that the non-dopaminergic system may also play a role in apathy.

A previous study reported that patients with PD presenting with apathy show the impairment of global cognitive function and diminished the ability to perform cognitive tasks (Pagonabarraga et al., 2015). In the current study, we focused on the effect of each cognitive subdomain on apathy. Apathy was significantly and independently associated with a decline in attention and executive functions, which was inconsistent with the results of a previous cross-sectional study (Pedersen et al., 2010) in which the authors did not observe any association between apathy and cognitive dysfunction, including the following cognitive domains: memory, attention/executive functions, psychomotor speed, and visuospatial skills. The association between apathy, attention, and executive dysfunction indicates possible underlying pathophysiological mechanisms that are common to these symptoms. A previous study that investigated apathy in patients with neurodegenerative conditions reported that patients with apathy showed impaired attention (Guimaraes et al., 2014). A prospective longitudinal study that included drug-naïve patients with PD (Santangelo et al., 2015b) also reported that baseline executive dysfunction was more severe in patients with PD presenting with apathy than in those without apathy, which suggests that poor performance on the Stroop test (which evaluates prefrontal cortex function) predicts the development of apathy after 2-years follow-up. Another study showed that repetitive transcranial magnetic stimulation improved the scores of patients' on the Stroop test, which suggests that executive and attention functions are associated with the frontal lobe activity (Boggio et al., 2005). A neuroimaging study (Benoit and Robert, 2011) showed that apathy in PD was associated with a reduction in gray matter volume or functional deficits in many regions, including the anterior and posterior cingulate cortices and dorsolateral or inferior frontal gyri. These findings suggest a strong association between apathy and prefrontal cortex dysfunctions, as identified in patients with other neurodegenerative diseases, such as Alzheimer's disease (Grossi et al., 2013). Moreover, the depletion of a cholinergic neuron has been implicated as an important contributor to cognitive dysfunction. This finding is supported by a double-blind, placebo-controlled study (Devos et al., 2014), which reported that rivastigmine could significantly improve the LARS score in patients with apathy but without dementia and depression. However, owing to the small number of patients who received the acetylcholinesterase inhibitor (AChE-I) treatment in our cohort, we did not investigate the effect of AChE-I on apathy in the current study.

Notably, men with PD were more likely to show apathy. A prospective longitudinal study (Wee et al., 2016) that included patients with PD observed that the progression of apathy was more rapid in men than in women. Our study indicates that apathy in PD could be considered a predictor of poor prognosis in men. However, the association between apathy and male sex should be considered with caution because apathy is more commonly observed in women than in men in the general population (Clarke et al., 2010). Further pathophysiological studies are warranted to verify the issue.

Another important finding in our study is that apathy was associated with depression scores, which is consistent with the results of two previous cross-sectional studies (Pedersen et al., 2010; Den Brok et al., 2015). It is reasonable to conclude that dysfunction of the dopaminergic mesocorticolimbic system, which is known to play a central role in the control of mood and motivation, is a common feature in both apathy and endogenous depression (Marin, 1991). A previous study in which PET (Maillet et al., 2016) was performed in patients with drug-naïve PD has proved the prominent role of serotonergic degeneration in the expression of apathy and depression. Conventionally, apathy is considered an aspect of depression; therefore, the association between depression scores and a high risk of apathy is not unexpected. Although apathy and depression are both commonly associated with PD (Den Brok et al., 2015), and both usually show overlapping symptoms (such as lack of interest), research has confirmed that apathy and depression can exist as distinct entities in patients with PD (Zahodne et al., 2012; Skidmore et al., 2013). However, since we found that apathy was not persistent in the current study, the predicted effect of depression on apathy is likely to be unstable and needs to be confirmed by further studies.

The limitations of this study are as follows: (A) The study did not include a group of healthy individuals (controls) against whom we could compare the progression of apathy in patients with PD. (B) This was a single-center study (all patients were recruited only from a single tertiary referral center in southwest China); therefore, our results should be confirmed by future multi-center studies. (C) Nearly 50% of the patients received baseline drug treatment; therefore, we could not verify the progression of “pure” apathy in patients with PD. (D) The relatively short period during which disease progression occurred in some patients is insufficient to determine the long-term evolution of apathy in PD. (E) We did not use specific instruments (or tools) to assess cognition. (F) Some patients did not reach the time to 4-years follow-up visit (*n* = 49), which contributed to the relatively small number of patients we included at that time (*n* = 52).

In conclusion, our study showed that the prevalence of apathy is higher in patients with progressive PD and that apathy is associated with male sex and disease-specific symptoms, including motor and non-motor symptoms. We observed that depression in early-stage PD is the main predictor of apathy in patients with PD. Our study highlights that apathy is an early, common, and non-persistent non-motor symptom in patients with early PD and that this finding may have implications for clinical management.

## Data Availability Statement

Data that support the findings of this study are available from the corresponding author (Huifang Shang, E-mail address: hfshang2002@126.com) upon reasonable request.

## Ethics Statement

The studies involving human participants were reviewed and approved by Ethics Committee of Sichuan University West China Hospital. The patients/participants provided their written informed consent to participate in this study.

## Author Contributions

RO, JL, and HS contributed to planning the study. RO contributed to data analysis and drafting of the manuscript. RO, JL, KL, ZJ, QW, YH, LZ, BC, BZ, and WS contributed to the recruitment of patients and processing of follow-up visits. All authors contributed to revising the manuscript.

## Conflict of Interest

The authors declare that the research was conducted in the absence of any commercial or financial relationships that could be construed as a potential conflict of interest.
