# Supplementary material for: Evolution of Apathy in Early Parkinson's Disease: A 4-Years Prospective Cohort Study
Source: Front Aging Neurosci. 2021 Jan 28;12:620762. doi: 10.3389/fnagi.2020.620762 (PMC7901914; doi:10.3389/fnagi.2020.620762)
Supplement: Supplementary file 1 [file Table_1.docx]

**Supplementary Table 1 Demographic and baseline clinical features between PD patients who completed the LARS assessment and those who did not or had missing data during follow up**

|  | Patients who were included in the study | Patients who were excluded in the study | *P*-value |
| --- | --- | --- | --- |
| Number of samples | 188 | 114 |  |
| Age, years, mean (SD) | 58.1 (10.7) | 60.2 (10.9) | 0.096 |
| Disease duration, mean (SD) | 1.5 (0.8) | 1.6 (0.9) | 0.102 |
| Male sex, n (%) | 95 (50.5) | 68 (59.6) | 0.123 |
| Education, mean (SD) | 10.7 (4.1) | 9.9 (4.0) | 0.097 |
| LEDD, mg/day, mean (SD) | 152.5 (188.1) | 186.6 (209.0) | 0.148 |
| Use of levodopa, n (%) | 74 (39.4) | 47 (41.2) | 0.748 |
| Use of dopamine agonist, n (%) | 46 (24.5) | 26 (22.8) | 0.743 |
| Use of anti-depressant, n (%) | 8 (4.3) | 4 (3.5) | 1.000 |
| FAB score, mean (SD) | 16.4 (1.9) | 16.1 (1.6) | 0.103 |
| MOCA score, mean (SD) | 25.5 (3.5) | 24.8 (3.0) | 0.055 |
| HDRS score, mean (SD) | 7.8 (7.2) | 8.8 (6.6) | 0.200 |
| HARS score, mean (SD) | 5.6 (5.5) | 6.1 (4.5) | 0.476 |
| UPDRS III score, mean (SD) | 23.3 (10.6) | 23.3 (10.1) | 0.958 |
| H&Y stage, mean (SD) | 1.9 (0.4) | 2.0 (0.5) | 0.342 |

Abbreviations: PD = Parkinson’s disease; LARS = Lille Apathy Rating Scale; LEDD = levodopa equivalent daily dose; FAB = frontal assessment battery; MOCA = Montreal Cognitive Assessment; HDRS = Hamilton Depression Rating Scale; HARS = Hamilton Anxiety Rating Scale; UPDRS = Unified Parkinson’s Disease Rating Scale.

**Supplementary Table 2 Demographic and baseline clinical features between PD patients who completed the 4-year follow-up and those who did not**

|  | With 4-year follow-up | Without 4-year follow-up | *P*-value |
| --- | --- | --- | --- |
| Number of samples | 52 | 136 |  |
| Age, years, mean (SD) | 59.0 (10.4) | 57.7 (10.9) | 0.458 |
| Disease duration, mean (SD) | 1.4 (0.8) | 1.6 (0.8) | 0.074 |
| Male sex, n (%) | 31 (59.6) | 64 (47.1) | 0.123 |
| Education, mean (SD) | 10.6 (4.0) | 10.8 (4.1) | 0.796 |
| LEDD, mg/day, mean (SD) | 144.9 (176.2) | 155.4 (197.3) | 0.736 |
| Use of levodopa, n (%) | 18 (34.6) | 56 (41.2) | 0.410 |
| Use of dopamine agonist, n (%) | 16 (30.8) | 30 (22.1) | 0.214 |
| Use of antidepressant, n (%) | 3 (5.8) | 5 (3.8) | 0.687 |
| FAB score, mean (SD) | 16.2 (1.9) | 16.5 (1.9) | 0.426 |
| MOCA score, mean (SD) | 25.2 (3.3) | 25.6 (3.5) | 0.440 |
| HDRS score, mean (SD) | 7.3 (7.3) | 7.9 (7.3) | 0.571 |
| HARS score, mean (SD) | 5.2 (5.5) | 5.8 (5.5) | 0.469 |
| UPDRS III score, mean (SD) | 22.5 (9.8) | 23.6 (11.0) | 0.535 |
| H&Y stage, mean (SD) | 2.0 (0.4) | 1.9 (0.4) | 0.318 |
| LARS score, mean (SD) | -25.2 (12.0) | -27.7 (9.7) | 0.152 |

Abbreviations: PD = Parkinson’s disease; LEDD = levodopa equivalent daily dose; FAB = frontal assessment battery; MOCA = Montreal Cognitive Assessment; HDRS = Hamilton Depression Rating Scale; HARS = Hamilton Anxiety Rating Scale; UPDRS = Unified Parkinson’s Disease Rating Scale; LARS = Lille Apathy Rating Scale.

**Supplementary Table 3 Comparisons of baseline characteristics between groups**

|  | At baseline |  |  | From baseline to 1-year | |  |
| --- | --- | --- | --- | --- | --- | --- |
|  | With apathy | Without apathy | *P*-value | With persistent apathy | With non-persistent apathy | *P*-value |
| Number of samples | 35 | 153 |  | 15 | 20 |  |
| Age, years, mean (SD) | 58.7 (12.6) | 57.9 (10.3) | 0.679 | 58.8 (14.3) | 58.7 (11.5) | 0.998 |
| Disease duration, years, mean (SD) | 1.7 (0.9) | 1.5 (0.8) | 0.297 | 1.3 (0.9) | 2.0 (0.8) | 0.021* |
| Male sex, n (%) | 22 (62.9) | 73 (47.7) | 0.106 | 10 (66.7) | 12 (60.0) | 0.686 |
| Education, years, mean (SD) | 10.1 (4.4) | 10.9 (4.0) | 0.302 | 11.0 (4.9) | 9.4 (4.0) | 0.282 |
| LEDD, mg/day, mean (SD) | 133.1 (170.4) | 148.6 (188.6) | 0.565 | 128.3 (154.9) | 136.7 (185.1) | 0.889 |
| Use of levodopa, n (%) | 12 (34.3) | 58 (37.9) | 0.689 | 5 (33.3) | 7 (35.0) | 0.918 |
| Use of dopamine agonist, n (%) | 7 (20.0) | 38 (24.8) | 0.545 | 3 (20.0) | 4 (20.0) | 1.000 |
| Use of antidepressant, n (%) | 3 (8.6) | 5 (3.3) | 0.169 | 2 (13.3) | 1 (5.0) | 0.565 |
| FAB score, mean (SD) | 15.6 (2.2) | 16.6 (1.7) | 0.005* | 15.5 (2.9) | 15.7 (1.7) | 0.842 |
| MOCA score, mean (SD) | 24.7 (3.8) | 25.7 (3.4) | 0.134 | 24.3 (4.4) | 25.0 (3.4) | 0.615 |
| HDRS score, mean (SD) | 11.9 (8.9) | 6.8 (6.5) | <0.001* | 13.3 (9.2) | 10.9 (8.7) | 0.442 |
| HARS score, mean (SD) | 8.4 (6.7) | 5.0 (5.0) | 0.007* | 9.7 (7.3) | 7.4 (6.2) | 0.313 |
| UPDRS III score, mean (SD) | 28.0 (12.3) | 22.3 (9.9) | 0.004* | 30.3 (10.5) | 26.2 (13.5) | 0.334 |
| H&Y stage, mean (SD) | 2.0 (0.4) | 1.9 (0.4) | 0.062 | 2.1 (0.3) | 2.0 (0.5) | 0.770 |

Abbreviations: LEDD = levodopa equivalent daily dose; FAB = frontal assessment battery; MOCA = Montreal Cognitive Assessment; HDRS = Hamilton Depression Rating Scale; HARS = Hamilton Anxiety Rating Scale; UPDRS = Unified Parkinson’s Disease Rating Scale. * Significant difference.

**Supplementary Table 4 Comparison of change in LEDD between patients with and without persistent apathy in PD**

|  | Patients with persistent apathy | Patients with nonpersistent apathy | *P*-value |
| --- | --- | --- | --- |
| Number of samples |  |  |  |
| From baseline to 1-year | 15 | 20 |  |
| From 1-year to 2-year | 17 | 20 |  |
| From 2-year to 3-year | 15 | 12 |  |
| From 3-year to 4-year | 11 | 3 |  |
| Change in LEDD, mg/day, mean (SD) | | |  |
| From baseline to 1-year | 201.7 (174.9) | 189.6 (145.2) | 0.825 |
| From 1-year to 2-year | 142.6 (131.6) | 94.4 (101.0) | 0.216 |
| From 2-year to 3-year | 170.0 (177.3) | 96.9 (156.9) | 0.273 |
| From 3-year to 4-year | 52.3 (95.2) | 8.3 (38.2) | 0.259 |

Abbreviations: PD = Parkinson’s disease; LEDD = levodopa equivalent daily dose.
