# Supplementary material for: Evolution of Apathy in Early Parkinson's Disease: A 4-Years Prospective Cohort Study
Source: Front Aging Neurosci. 2021 Jan 28;12:620762. doi: 10.3389/fnagi.2020.620762 (PMC7901914; doi:10.3389/fnagi.2020.620762)
Supplement: Supplementary file 2 [file Image_1.pdf]

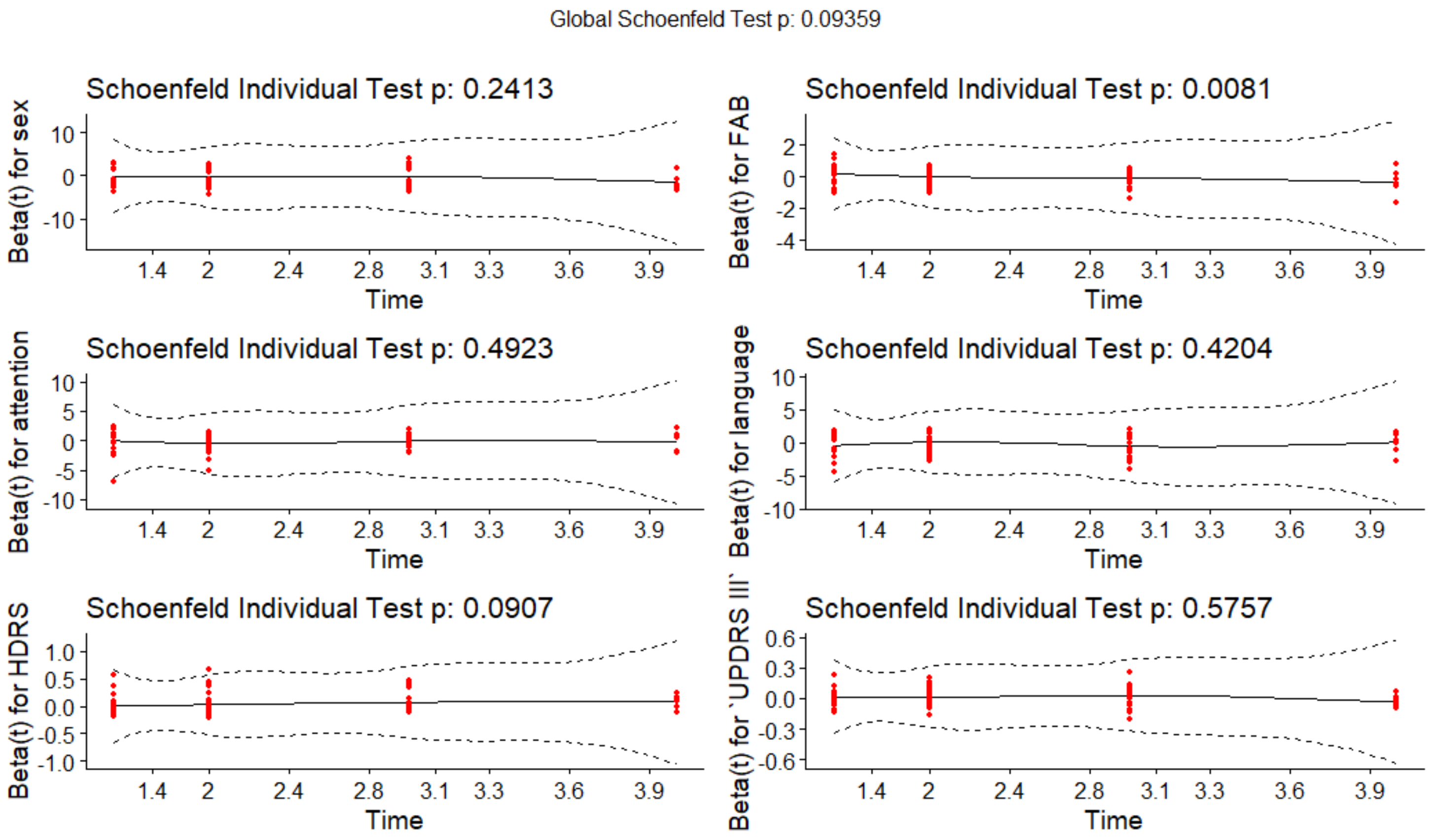

Supplementary Figure 1 Test for the proportional hazard assumption for Cox model

The Schoenfeld individual test indicated that the Schoenfeld had no significant relationship with time ($P = 0.094$), suggesting that the Cox model met the proportional hazard assumption.
